# Touching soft materials slows affective visual processing

**DOI:** 10.3389/fpsyg.2025.1644393

**Published:** 2025-11-26

**Authors:** Achille Pasqualotto, Utek Leong, Ryo Kitada

**Affiliations:** 1 School of Social Sciences, Nanyang Technological University, Singapore, Singapore; 2 Faculty of Human Sciences, University of Tsukuba, Tsukuba, Japan; 3 Japan Society for the Promotion of Science (JSPS), Tokyo, Japan; 4 Department of Psychology, National University of Singapore, Singapore, Singapore; 5 Graduate School of Intercultural Studies, Kobe University, Kobe, Japan

**Keywords:** multisensory integration, vision, touch, valence, affective processing

## Abstract

Presently, there is extensive evidence of multisensory integration in tactile and visual processing. While it has been shown that multisensory interaction between touch and vision influences many cognitive processes, such as object recognition, the role of multisensory interaction in the affective domain is still poorly understood. The aim of this study was to examine the influence of tactile perception on the affective processing of visual stimuli. Two experiments were conducted with urethane rubbers of differing compliance and with visually presented words. In the first experiment, participants rated the affective valence of the visually presented words while touching hard or soft urethane rubbers. Ratings and reaction times were recorded. Results showed touching a soft stimulus slowed the valence rating of visual words, but it did not affect the valence ratings per se. A second experiment clarified whether this effect was unique to valence (affective) ratings or whether it extended to semantic (cognitive) ratings as well. The second experiment was identical to the first one, but here participants rated the level of abstractness of the same visually presented words. Results indicated that abstractness ratings were not affected by the tactile stimuli. Overall, these confirm that, possibly via an attentional mechanism, tactile input influences the speed of affective visual processing.

## 1 Introduction

In our daily lives, we tend to think of our senses as distinct modalities that individually afford the perception of the objects around us. However, we often disregard how our senses work together to enhance our perceptions and how important this integration is in our daily lives (Stein, 2012).

Of particular interest is the interaction between vision and touch, which encompasses object recognition (Klatzky and Lederman, 2011; Lacey and Sathian, 2011; Martinovic et al., 2012; Newell et al., 2001), object localization (Cattaneo and Vecchi, 2008; Pasqualotto et al., 2013; Woods et al., 2004; Zuidhoek et al., 2004), as well as body representation (Della Longa et al., 2022; Kaneno et al., 2024; Pasqualotto and Proulx, 2015). Indeed, these two modalities complement one another by providing us with distinct sets of information (e.g., color by vision and temperature by touch). Therefore, multisensory integration between vision and touch has been broadly investigated (e.g., Pasqualotto et al., 2016), yet the affective component of visuo-tactile integration has been far less studied and understood (Filippetti et al., 2019; Morrison, 2016). We focused on touch because it is well-suited for affective processing (e.g., Cavdan et al., 2023; Drewing et al., 2018; Kirsch et al., 2020; Guest et al., 2011; Pasqualotto et al., 2020). For example, our studies found evidence that soft tactile stimuli engender pleasantness (Pasqualotto et al., 2020), and softness perception activates the insula, a region of the brain involved in affective processing (Kitada et al., 2019).

Previous studies suggested that perceiving pleasurable attributes in one sensory modality (e.g., touch) affects the overall multisensory experience of a product by biasing affective perceptions of other sensory modalities (e.g., vision and audition) (see Spence, 2022; Spence and Gallace, 2011 for reviews). Suzuki and Gyoba (2008) found that the repeated visual exposure to novel stimuli (mere-exposure effect, Zajonc, 1968) increased the participants’ subsequent preference for those stimuli when judged by touch. Wu et al. (2011) exposed participants to multisensory (visual and tactile) stimuli and found a similar mere-exposure effect for affective judgements within the visual domain. Are the results of these studies examples of multisensory effective priming? Although unisensory (vision), the study by Pecchinenda et al. (2014) offers an interesting methodology. In their Experiment 1, symmetric/asymmetric ‘clouds’ of dots preceded words that participants categorized as positive or negative. Authors found that symmetric clouds improved the categorization of positive words. This study belongs to the vast literature on affective visual priming, reporting congruency effects between a priming stimulus and the response of the observers (both in terms of accuracy and reaction times) (Fazio, 2001; Hermans et al., 1994, 2001). Here, symmetric clouds were perceived as more pleasant (Friedenberg and Bertamini, 2015), thus improving the processing of positive words (congruency). Would the same results stand in a multisensory setting?

We decided to investigate the effect of tactile stimuli on affective visual processing adapting the affective priming used by Pecchinenda et al. (2014), but utilised urethane rubbers of different compliance (soft/hard) as tactile stimuli (rather than clouds of dots), and words of varied valence (positive/neutral/negative) as visual stimuli, which participants rated in terms of valence. These urethane rubbers have been used in other experiments of ours experiments of yours (e.g., Pasqualotto et al., 2020), and we know their physical characteristics and pleasantness. To our knowledge, no study has investigated affective priming between tactile softness and words of valence.

We expect that the characteristics of the tactile stimuli (soft/hard) will interact with the valence of the visual stimuli (positive/neutral/ negative), thus influencing participants’ affective judgement of the visually presented word.

## 2 Experiment 1

The first experiment tested our principal hypothesis about the effect of touch on affective visual processing. Here, participants evaluated the valence of words presented on a computer screen, *while* they were touching hard/soft urethane rubbers. Beforehand, a pilot study helped us to choose and classify the visual words.

### 2.1 Materials and methods

#### 2.1.1 Participants

Twenty-four (12 male and 12 female) right-handed individuals aged between 19 and 32 (mean 23.42 years) participated to Experiment 1 and were recruited via posters placed around Nanyang Technological University’s campus. The minimum sample size was determined by the experiments on affective priming presented by Pecchinenda et al. (2014). Participants’ handedness was obtained using the Fazio Laterality Inventory (FLI; Fazio et al., 2013). Participants did not present any tactile impairments or injuries on their hands and had normal or corrected-to-normal vision. All of them provided written informed consent before starting the experiment (or the pilot, see below). All studies were approved by the Institutional Review Board (IRB-2018-07-013) at Nanyang Technological University, thus are in accordance with the ethical standards of the Helsinki Declaration of 1975, as revised in 2000. Participants received 10 Singaporean Dollars (SGD) for their participation (5 SGD for the pilot).

#### 2.1.2 Apparatus

In order to select the visual stimuli for Experiment 1, a pilot study (*N* = 10; five male and five female, average age 23.6 years, with normal or corrected-to-normal vision) was run using 130 words from the Affective Norms for English Words (ANEW; Bradley and Lang, 1999) and the software Presentation™ (Berkeley, USA). Participants were randomly presented with these words twice; during one presentation, participants rated the *Valence* of the words (from “not positive at all” to “very positive”) on a scale from 1-to-9 (with 1 indicating “not positive at all”), while in the other presentation participants rated how much a word was associated with tactile sensations (from “not associated at all” to “very associated”) on a scale from 1-to-9 (with 1 indicating “not associated at all”). For example, the word “book” would have a Tactile Association score higher than the word “cloud.” Knowing the Tactile Association ratings was necessary to ensure that all the selected words had small and comparable pre-existing associations with tactile sensations, thus preventing potential confounds in Experiment 1. The tasks’ order (Valence rating and Tactile Association rating) was counterbalanced across participants.

The average Valence and Tactile Association ratings were calculated for each word and then words were ordered by their Valence. Then we selected the top eight words (i.e., those with the highest Valence, or Positive Words, e.g., “Free”), the bottom eight words (those with the lowest Valence, or Negative Words, e.g., “Death”), and the eight words that were around the average Valence rating (5.72), or Neutral Words (e.g., “Farm”). At the same time, to avoid biases, the selected words had to have Tactile Association ratings below the average Tactile Association (4.53). The selected words (see Supplementary Table S1 for details) were visually presented in Experiment 1.

Four polyurethane rubbers (Katō Tech, Kyoto, Japan) with differing compliance were used in Experiment 1; rubber A (compliance: 0.13 mm/N), rubber B (0.45 mm/N), rubber H (7.56 mm/N), and rubber I (10.53 mm/N). Compliance indicates rubbers’ indentation when the same pressure is applied [refer to Pasqualotto et al. (2020) for a detailed explanation of the compliance measurement]. Therefore, as indicated by their low compliance values, rubbers A and B were the “Hard” rubbers, while H and I where the “Soft” rubbers. Additionally, based to our previous study (Pasqualotto et al., 2020), we know that soft rubbers (H and I) were more pleasant than hard rubbers (A and B). To ensure that rubbers were presented in the same manner, we used the device called Model SHR III-5 SK (Aikoh Engineering, Osaka, Japan) to press the rubbers onto three fingers of the participants at the same speed (5 cm/s) and with the same maximum applied force (5 N). This device was used in our previous studies (e.g., Pasqualotto et al., 2020).

#### 2.1.3 Procedure

**Figure 1:**
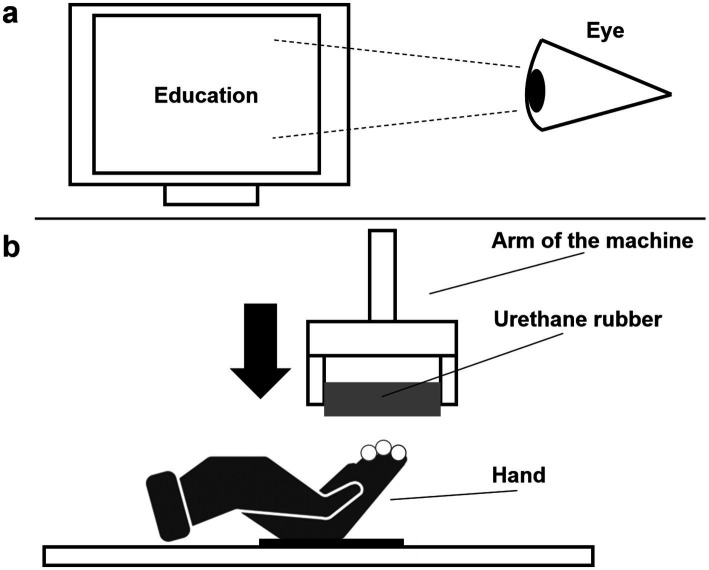
Experimental setup: **(a)** the monitor showing the 24 visual stimuli (in this case, the word “Education”), while *at the same time* **(b)** the arm of the machine lowers to present the four tactile stimuli to the gloved hand fixed with Velcro to the table; the fingertips of three fingers (index, middle, and ring) are exposed to the tactile stimulus (the image of the hand was designed by Freepik [https://www.freepik.com] and modified for this article).

Upon giving informed written consent, completing the handedness questionnaire (FLI), and a brief demographic questionnaire, participants sat in front of the apparatus (see Figure 1 for details). Words were presented on a computer screen (36 × 28 cm, positioned about 35 cm apart) and, to ensure that participants paid attention to the *visually* presented words they rested their heads on an ophthalmic chin rest. Words subtended a visual angle included between 8.99° and 3.27° width (depending in words’ length) and 2.46° height. Instructions were: “Please, rate the words that will appear on the screen in terms of how positive they are. Use a scale from 1-to-9, with 1 = “not positive at all” and 9 = “very positive.” Participants used their non-dominant (left) hands to type their answers on a keypad. They were asked to pay attention only to the words appearing on the screen placed in front of them and to ignore tactile stimuli. Participants were required to answer as quickly and as accurately as possible.

Words remained on the screen until an answer was produced or up to 2,500 ms. The tactile stimuli remained in touch with participants’ hands until the applied force of 5 N was reached. The initial five trials were for practice only, and employed rubbers and visuals words that were not used in the actual experiment. Twenty-four experimental trials were then conducted for each participant. The visually presented words were pseudo-randomized such that words belonging to the same category (positive/neutral/negative) were never consecutively presented. Likewise, tactile stimuli of the same category (soft/hard) were never consecutively presented. Valence ratings and reaction times (RT) were recorded for each trial. The experimental session lasted about 30 min, while the pilot experiment lasted about 15 min. None of the participants who joined the pilot were allowed to take part in Experiment 1.

### 2.2 Results

For each participant we calculated the average Valence Ratings and RT (dependent variables) for the three types of Visual Words (Positive/Neutral/Negative, first independent variable) and two types of Tactile Rubbers (Soft/Hard, second independent variable), thus resulting in a 3 × 2 design. These data underwent statistical processing.

#### 2.2.1 Valence ratings

The Shapiro–Wilk test of normality was significant for two-out-of-six datasets [*W*(24) = 0.885, *p* = 0.011 and *W*(24) = 0.896, *p* = 0.017, while for all the others *W*(24) > 0.957, *p* > 0.387]. Since most of the tests was not significant (4-out-of-6) then we conducted parametric statistical tests on our data (Field, 2009).

We ran a two-way within-subjects ANOVA on the average Valence Ratings with Visual Words and Tactile Rubbers as factors. The Mauchly’s test showed that the assumption of sphericity was violated for the main effect of Visual Words [*χ^2^*(2) = 7.22, *p* = 0.030]. Therefore, the associated degree of freedom for the main effect of Visual Words was corrected using Greenhouse–Geisser estimates of sphericity.

**Figure 2:**
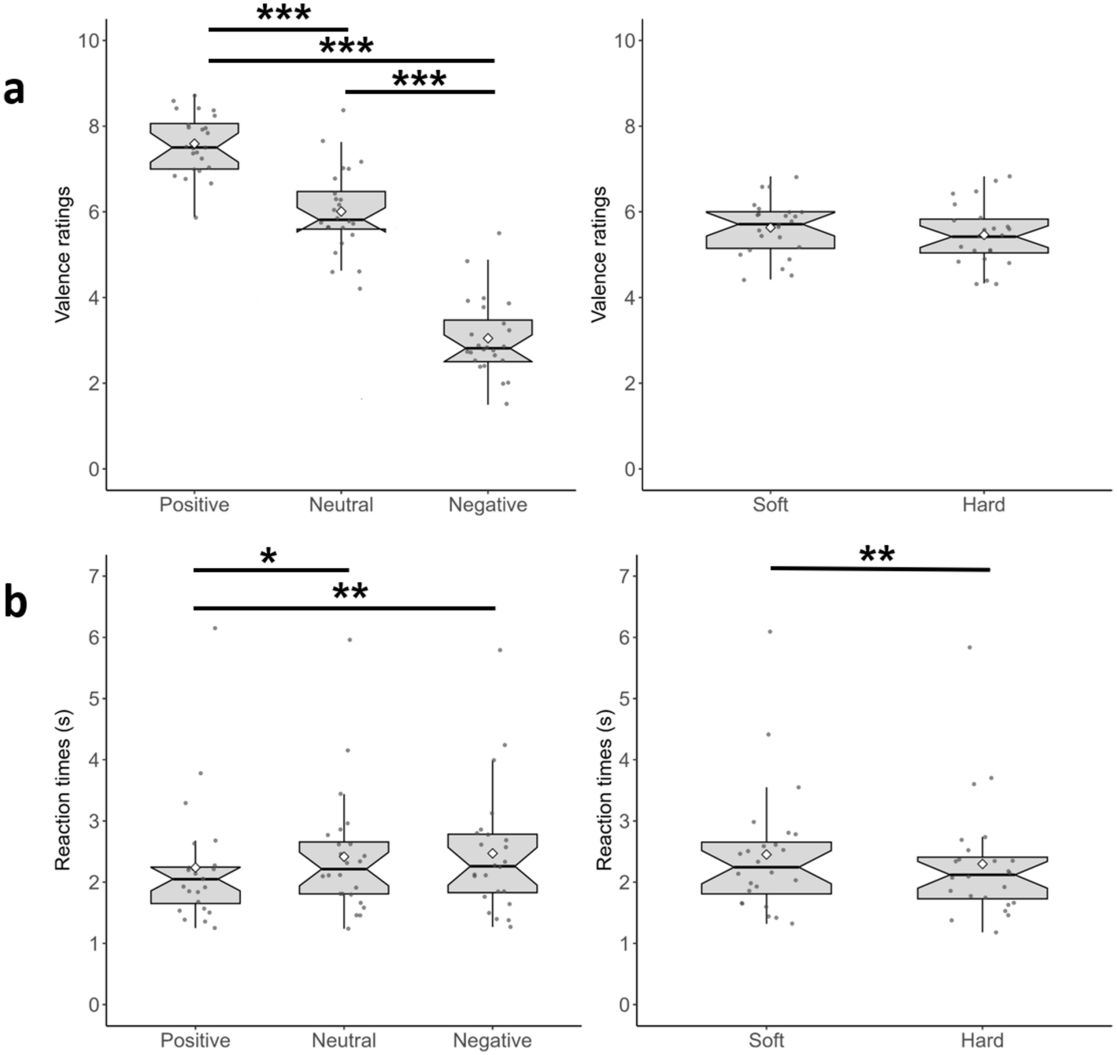
Results of Experiment 1 on Valence Ratings and RT; the size of the bars indicates the quartile, the horizontal lines inside each bar indicate the median, and the white diamonds indicate the average. Whiskers represent the largest and smallest data values that are within 1.5 × the interquartile range (IQR) above the third quartile (Q3) and below the first quartile (Q1), respectively. The dots above/below the whiskers are the outliers. The notches of the boxes indicate the 95% confidence level around the median. **(a)** Valence Ratings for the Visual Words (left) and Tactile Rubbers (right); **(b)** RT for the same variables. ***p < 0.001, **p < 0.010, and *p < 0.050.

The results of the two-way ANOVA showed a statistically significant main effect of Visual Words on participants’ Valence Ratings [*F*(1.56, 35.94) = 171.98, *p* < 0.001, *η_p_^2^* = 0.88]. Post-hoc pairwise comparisons with Bonferroni adjustment indicated that the average Valence Ratings were significantly higher for Positive Visual Words (*M* = 7.59, SD = 0.90) than for Neutral Visual Words (*M* = 6.01, SD = 1.15) [*p* < 0.001] and Negative Visual Words (*M* = 3.04, SD = 1.20) [*p* < 0.001]. Average Valence Ratings were also significantly higher for Neutral Visual Words than for Negative Visual Words [*p* < 0.001] (see Figure 2a, left). This confirms the words’ categorization that emerged from the pilot experiment.

The main effect of Tactile Rubbers and the interaction between Visual Words and Tactile Rubbers were not statistically significant [*F*(1, 23) = 0.87, *p* = 0.36, *η_p_^2^* = 0.04 and *F*(2, 46) = 1.78, *p* = 0.18, *η_p_^2^* = 0.07, respectively] (see Figure 2a, right). This suggests that the valence ratings of visually presented words were not influenced by the tactile stimuli.

#### 2.2.2 Reaction times

The Shapiro–Wilk test of normality was significant for each dataset [all *W*(24) < 0.907, all *p* < 0.031]. Therefore, we conducted non-parametric statistical tests on our data.

Since our design requires a two-way ANOVA, to clarify the main effects of Visual Words and Tactile Rubbers we averaged these variables. The first procedure produced three datasets based on the types of Visual Word, which were analyzed using a Related-Samples Friedman test. Results showed a significant effect of the Visual Words [χ^2^(5) = 12.33, *p* = 0.002], and the Bonferroni-corrected pairwise comparisons confirmed that Positive Visual Words were rated more rapidly (*M* = 2.24 s, SD = 1.03) than Neural (*M* = 2.42 s, SD = 1.03) and Negative (*M* = 2.47 s, SD = 1.06) Visual Words [*p* = 0.012 and *p* = 0.004, respectively] (see Figure 2b, left). This indicates that positive words were rated more rapidly. Then, we averaged RT across the three types of Visual Words to obtain two datasets based on the types of Tactile Rubbers, which were analyzed using a Related-Samples Wilcoxon Test. Results showed a significant difference [*Z* = 34.50, *p* < 0.002], thus confirming that touching Soft rubbers slowed RT (*M* = 2.45 s, SD = 1.02 s), compared to Hard rubbers (*M* = 2.30 s, SD = 1.02 s) (see Figure 2b, right). Finally, investigate the interaction between Visual Words and Tactile Rubbers, for each visual condition (Positive/Neutral/Negative) we calculated the difference Soft minus Hard (see Supplementary Table S3 for details) and ran a Related-Samples Friedman test. The result was not significant [χ^2^(2) = 0.333, *p* = 0.846], indicating the lack of interaction.

### 2.3 Discussion

The main results of Experiment 1 were that (1) words with a positive valence were faster to rate. Additionally, (2) touching hard rubbers speeded the rating of *all* the visually presented words. Although the advantage for processing words with a positive valence is still strongly debated (Kauschke et al., 2019), most of the evidence favours the notion of faster reaction times for positive words during affective processing (Kappes and Bermeitinger, 2016; Kever et al., 2017; Liu et al., 2016; Stenberg et al., 1998). Therefore, our first result (faster processing of positive words) confirms this major trend in literature.

The other main finding was that hard tactile stimuli speeded the rating of visual words or, that soft tactile stimuli *slowed* the rating of visual words. We favor the latter interpretation of the results, because our past research (Pasqualotto et al., 2020) showed that the soft stimuli used in this experiment are also more pleasant. Although somewhat debated (Luck et al., 2021), pleasant stimuli are likely to attract more attention (Gupta, 2019) and to slow the reaction times for visually presented words. If this is the case, would pleasant tactile stimuli affect any kind of visual processing? Or just the visual processing involving affective valence? Evidence for the latter would suggest task-specific interactions between touch and vision (Lacey and Sathian, 2011; Pascual-Leone and Hamilton, 2001; Pasqualotto et al., 2013). To clarify this, we ran Experiment 2.

## 3 Experiment 2

Experiment 2 investigated the multisensory interaction of touch and vision, but using a semantic task that, unlike Experiment 1, did not involve the affective component. Specifically, here participants rated the level of abstractness of the same visually presented words used in Experiment 1, while they were touching the same urethane rubbers. A pilot study helped us to classify the visual words into three levels of abstractness (High/Medium/Low).

### 3.1 Materials and methods

#### 3.1.1 Participants

Twenty-four (12 male and 12 female) naïve right-handed volunteers aged between 18 and 57 (mean 26.04 years) participated to Experiment 2. All the other details were the same as in Experiment 1.

#### 3.1.2 Apparatus

In order to classify the visual stimuli for Experiment 2, a pilot study (*N* = 10; five male and five female, average age 25 years) was conducted. Participants were randomly presented with the 24 visual words used in Experiment 1, and they rated the level of *abstractness* of these words (from “not abstract at all” to “very abstract”) on a scale from 1-to-9 (with 1 indicating “not abstract at all”).

Average Abstractness ratings were calculated for each word and then words were ordered by their Abstractness. The top eight words (i.e., those with the highest abstractness ratings) were classified as High, the bottom eight words (those with the lowest abstractness ratings) were classified as Low, and the eight words in the middle were classified as Medium (see Supplementary Table S2 for details). All the other details were identical to Experiment 1.

#### 3.1.3 Procedure

The procedure of Experiment 2 was the same as Experiment 1, with the exception that participants received the instruction: “Please, rate the words that will appear on the screen in terms of how abstract they are. Use a scale from 1-to-9, with 1 = “not abstract at all” and 9 = “very abstract.”

### 3.2 Results

Raw data were pre-processed as in Experiment 1.

#### 3.2.1 Valence ratings

The Shapiro–Wilk test of normality was significant for one-out-of-six datasets [*W*(24) = 0.905, *p* = 0.028, while for all the others *W*(24) > 0.941, *p* > 0.174]. Therefore, we conducted parametric statistical tests on our data.

As in Experiment 1, a two-way within-subjects ANOVA on the Visual Words average ratings with Visual Words (High Abstractness/Medium Abstractness/Low Abstractness) and Tactile Rubbers (Hard vs. Soft) as independent variables was run.

The Mauchly’s test showed that the assumption of sphericity was violated for the main effect of Visual Words [*χ^2^*(2) = 11.44, *p* = 0.030]. Therefore, for this variable the degree of freedom was corrected using Greenhouse–Geisser estimates of sphericity.

**Figure 3:**
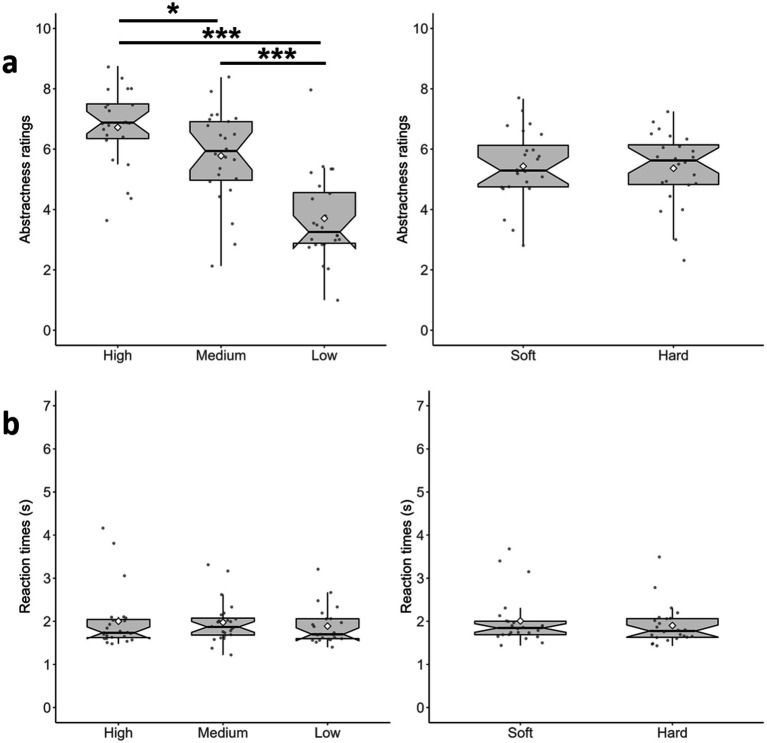
Results of Experiment 1 on Abstractness Ratings and RT; the size of the bars indicates the quartile, the horizontal lines inside each bar indicate the median, and the white diamonds indicate the average. Whiskers represent the largest and smallest data values that are within 1.5 × the interquartile range (IQR) above the third quartile (Q3) and below the first quartile (Q1), respectively. The dots above/below the whiskers are the outliers. The notches of the boxes indicate the 95% confidence level around the median. **(a)** Abstractness Ratings for the Visual Words (left) and Tactile Rubbers (right); **(b)** RT for the same variables. ***p < 0.001, **p < 0.010, and *p < 0.050.

The results of the two-way ANOVA highlighted a statistically significant main effect of Visual Words on the participants’ abstractness ratings [*F*(1.42, 32.73) = 44.97, *p* < 0.001, *η_p_^2^* = 0.660]. Post-hoc pairwise comparisons with Bonferroni adjustments indicated that the average abstractness ratings were significantly higher for High Abstractness words (M = 6.72, SD = 1.43) than for Medium Abstractness words (*M* = 5.78, SD = 1.71) [*p* < 0.017] and Low Abstractness words (*M* = 3.70, SD = 1.65) [*p* < 0.001]. Average abstractness ratings were also significantly higher for Medium Abstractness words than for Low Abstractness Words [*p* < 0.001]. Substantially, these results corroborate the categorization of the words that emerged from the pilot (see Figure 3a, left).

The main effect of Tactile Rubbers and the interaction effect between Visual Words and Tactile Rubbers were not statistically significant [*F*(1, 23) = 0.12, *p* = 0.735, *η_p_^2^* = 0.010 and *F*(2, 46) = 0.76, *p* = 0.474, *η_p_^2^* = 0.030, respectively] (see Figure 3a, right). These results suggest that abstractness ratings are mostly influenced by the abstractness of the displayed words.

#### 3.2.2 Reaction times

The Shapiro–Wilk test of normality was significant for five-out-of-six datasets [all *W*(24) < 0.883, all *p* < 0.009, while for one dataset it was not *W*(24) = 0.937, *p* = 0.143]. Therefore, we conducted non-parametric statistical tests on our data.

As in Experiment 1, for the main effects of Visual Words and Tactile Rubbers we averaged these variables. A Related-Samples Friedman test showed a lack of significant effect of the Visual Words [χ^2^(2) = 1.36, *p* = 0.506], see Figure 3b, left. Then, a Related-Samples Wilcoxon Test indicted a non-significant effect for the Tactile Rubbers (*Z* = 105.50, *p* = 0.203, see Figure 3b, right). Finally, to investigate the interaction between Visual Words and Tactile Rubbers, for each visual condition (High/Medium/Low) we calculated the difference Soft minus Hard (see Supplementary Table S4 for details) and ran a Related-Samples Friedman test., which was not significant [χ^2^(2) = 0.583, *p* = 0.747].

### 3.3 Discussion

The aim of Experiment 2 was to better characterize the results of Experiment 1. We found that touching soft stimuli did *not* significantly affect the speed of abstractness ratings relative to words presented through vision. This suggests that the cross-modal interaction between touch and vision is task-sensitive (e.g., Lacey and Sathian, 2011) and can be measured in tasks involving the affective component only (Experiment 1).

## 4 General discussion

Our study investigated the multisensory interaction of vision and touch within the affective domain. Initially we found that the presentation of tactile stimuli did not affect the valance ratings of visual words, yet soft tactile stimuli slowed the affective valence ratings for visually presented words, compared to hard tactile stimuli. Then we better characterized the above results in terms of task-specificity; touching soft stimuli slowed the rating of visually presented words *only* in affective ratings (and not in semantic ratings). This set of results suggests touch affects the speed of affective visual processing.

Pecchinenda et al. (2014) reported an interaction between the valence of the words and the symmetricity of the dot clouds in terms of accuracy (but not in terms of reaction times). Yet, unlike them, we did not find an interaction between the valence of the words and the softness of the rubbers, but instead a *main* effect of softness in terms of reaction times. Our lack of interaction can be largely explained by the fact that their study was unisensory (vision) while ours was multisensory (vision and touch). Indeed, each sensory modality represents information in slightly different manners (Behrmann and Ewell, 2003; Lacey et al., 2007; Whitaker et al., 2008), therefore the representation of visually perceived affective stimuli is partly different from tactile perceived affective stimuli, and thus in our case interaction in the valence evaluation was not observed. Nevertheless, through different sets of results, both studies reported the effect of affective stimuli on affective processing, but not on semantic processing.

Studies on *visual* priming (including Pecchinenda and colleagues), where a visual stimulus facilitates the processing of the following visual stimulus, reported that the type of judgement required to participants (semantic vs. affective) determined the presence/absence of the priming itself (Klauer and Musch, 2002; Lichtenstein-Vidne et al., 2012; Spruyt et al., 2007; Storbeck and Robinson, 2004). Thus, semantic priming would occur when stimuli undergo a semantic judgement (e.g., is it a verb or an adjective?), while affective priming would occur when the same stimuli undergo an affective judgement (e.g., is it good or bad?). Indeed, in our experiments we found an effect of the tactile stimuli *only* when participants were asked to perform the affective judgement of the visual stimuli. Therefore, in terms of task-specificity our results are in line with previous research involving the visual modality and extend our knowledge to the vision-tactile (multisensory) domain.

Although we found that touching soft stimuli slows the affective processing of visually presented words, and this result fits with the priming literature, we are aware that in our experiments tactile and visual stimuli were presented at the same time, thus representing a particular case of priming with no interstimulus interval. Additionally, it is not entirely clear whether soft tactile stimuli slowed the affective judgment of visual stimuli or whether *hard* tactile stimuli *speeded* the affective judgment of visual stimuli. Yet, our previous research found that tactile stimulation of softer objects felt more pleasant (Kitada et al., 2021; Pasqualotto et al., 2020), thus supporting the interpretation that, probably through an attentional mechanism, soft tactile stimuli slowed the rating of visual words. The modulation of attention by soft-and-pleasant stimuli is also supported by Kawamichi et al. (2015), who reported that touching a friend’s hand (relative to a non-embodied rubber hand, Fahey et al., 2019) reduced the unpleasantness of aversive visual stimuli and reduced visual cortex activity. This suggests that the more pleasant (“comfortable”) tactile stimulation swayed the attention from the visual stimuli. Yet, although speculative, both processes might be at play, with soft objects slowing the affective judgment of visual stimuli via the above-mentioned attentional mechanism and hard objects speeding their judgement.

We found that the link between touch and vision was limited to the affective processing, thus task-sensitive. This is a rather established finding (Ghazanfar and Schroeder, 2006; Pascual-Leone and Hamilton, 2001), where “unimodal” and “associative” brain areas cooperate to accomplish the same task. Likewise, we found that an affective task carried out by vision was influenced by a tactile input eliciting pleasantness. Future studies should address how the present results could extend to other stimuli, such as textures (Roberts et al., 2024), and affect stimuli selection (Streicher and Estes, 2016).

In conclusion, although the generalization of our results should be tested using images rather that words (Bradley and Lang, 2007), our study for the first time investigated the effect of tactile input on the affective judgment of visual stimuli and found that softness slows judgment, and that this cross-modal effect is task-specific.

## Data Availability

Data are available at: https://osf.io/86der/overview.
