# Supplementary material for: Touching soft materials slows affective visual processing
Source: Front Psychol. 2025 Nov 26;16:1644393. doi: 10.3389/fpsyg.2025.1644393 (PMC12690938; doi:10.3389/fpsyg.2025.1644393)
Supplement: Supplementary file 1 [file Supplementary_file_1.docx]

Touching soft materials slows affective visual processing

Supplementary materials

Achille Pasqualotto^1 2 3^ *, Utek Leong^1 4^, and Ryo Kitada^1 5^

^1^ School of Social Sciences, Nanyang Technological University, Singapore

^2^ Faculty of Human Sciences, University of Tsukuba, Japan

^3^Japan Society for the Promotion of Science (JSPS), Tokyo, Japan

^4^ Department of Psychology, National University of Singapore, Singapore

^5^ Graduate School of Intercultural Studies, Kobe University, Japan

Table S1: Visually presented words in Experiment 1 (in alphabetical order).

| Positive  Words | Average  Valance | Neutral  Words | Average  Valance | Negative  Words | Average  Valance |
| --- | --- | --- | --- | --- | --- |
| Education | 7.8 | Blue | 6 | Alone | 2.8 |
| Free | 7.8 | Doctor | 5.9 | Death | 2.7 |
| Health | 7.7 | Farm | 5.9 | Fear | 2.7 |
| Hope | 7.8 | Manner | 5.8 | Fire | 4.2 |
| Justice | 7.8 | Market | 5.8 | Industry | 3.5 |
| Kind | 7.6 | Mind | 6 | Lost | 3.6 |
| Life | 7.6 | Power | 6 | Stress | 3 |
| Peace | 7.5 | Theory | 5.4 | Trouble | 3.5 |

Table S2: Visually presented words in Experiment 2 (in alphabetical order).

| High  Abstractness words | Average  Abstractness | Medium  Abstractness words | Average  Abstractness | Low  Abstractness words | Average  Abstractness |
| --- | --- | --- | --- | --- | --- |
| Death | 7 | Alone | 6.2 | Blue | 2 |
| Fear | 7.3 | Education | 5.5 | Doctor | 2.3 |
| Free | 6.7 | Health | 5.3 | Farm | 2.5 |
| Hope | 6.8 | Lost | 6 | Fire | 2.3 |
| Justice | 7.6 | Mind | 5.8 | Industry | 2.9 |
| Life | 6.7 | Power | 6 | Kind | 5.1 |
| Peace | 6.8 | Stress | 5.9 | Manner | 4.3 |
| Theory | 7.6 | Trouble | 5.3 | Market | 3.2 |

Table S3: Reaction times differences between Soft and Hard conditions in Experiment 1

| Difference | Average | Standard deviation |
| --- | --- | --- |
| (Soft-Positive) – (Hard-Positive) | 1.33 | 3.32 |
| (Soft-Neutral) – (Hard-Neutral) | 1.02 | 4.73 |
| (Soft-Negative) – (Hard-Negative) | 2.30 | 4.93 |

Table S4: Reaction times differences between Soft and Hard conditions in Experiment 2

| Difference | Average | Standard deviation |
| --- | --- | --- |
| (Soft-High) – (Hard-High) | 1.89 | 4.72 |
| (Soft-Medium) – (Hard-Medium) | 1.95 | 5.44 |
| (Soft-Low) – (Hard-Low) | 1.86 | 5.25 |
